# Biaxial tensile test and meso damage numerical simulation of HTPB propellant

**DOI:** 10.1038/s41598-022-22726-8

**Published:** 2022-10-21

**Authors:** Qizhou Wang, Guang Wang, Zhejun Wang, Hongfu Qiang, Xueren Wang, Shiqi Li, Zhaojun Zhu

**Affiliations:** 1 Rocket Force University of Engineering, Xi’an, 710025 China; 2 Zhengzhou University of Aeronautics, Zhengzhou, 450015 China

**Keywords:** Aerospace engineering, Mechanical properties

## Abstract

Aiming at the shortcomings of the current research on the mechanical properties of solid propellants under complex stress conditions, an effective cross-shaped test piece configuration and variable-scale biaxial tensile test method are designed in this paper, and the meso-simulation model of propellant is constructed by Micro-CT test and random filling algorithm. Then, based on the Hook-Jeeves method and the cohesive force model, the mechanical performance parameters of each mesoscopic component were obtained, and finally the damage evolution process of the propellant was numerically simulated. The results show that the stress–strain curve of the propellant under biaxial loading is similar to that of uniaxial stretching, and has obvious rate dependence and stress state dependence. The mechanical properties of the propellant under biaxial tensile loading are significantly lower than those in uniaxial stretching, and the maximum elongation is only 45–85% of that in uniaxial stretching. The fracture process of propellant can be divided into initial linear stage, damage evolution stage and fracture stage. The dewetting phenomenon generally occurs at the interface between the large-sized AP particles and the matrix. With the loading of the load, the pores formed by the dewetting and matrix tearing continue to converge into cracks and expand in the direction perpendicular to the resultant force, and finally fracture. The propellant dehumidifies more easily under high strain rate loading, but the degree of dewetting is lower when the same strain is reached.

## Introduction

Solid propellant is the power source of solid rocket motor (SRM), and its mechanical properties directly affect the carrying capacity of SRM^1^. At present, most of the research on the mechanical properties of solid propellants is based on uniaxial tensile tests. However, in the whole life cycle of the SRM grain, complex stress states such as biaxial tension, biaxial compression, and biaxial tension and compression will appear, not just a simple uniaxial force state^2^. Therefore, the mechanical behavior of solid propellant under one-dimensional stress state cannot effectively verify the structural integrity of SRM^3^, and it is necessary to carry out research on the mechanical properties of solid propellant under complex stress state. Studies^4,5^ have shown that the most prone to failure and instability of the grain is the inner hole surface under normal circumstances. Especially at the moment of SRM ignition, the superimposed loads such as the external environment and internal pressure can affect the inner hole surface of the grain column, which approximates the biaxial tensile load^6^.

In order to study the mechanical behavior of solid propellant under biaxial tensile load, Bills^7^, Wang^8^ carried out a biaxial tensile mechanical performance test of solid propellant with strip-shaped test pieces, and applied the data to the engine in troubleshooting. Liu C^9^ and Zhao W C^10^ studied the biaxial tensile mechanical properties of propellants after thermal aging by using strip-shaped specimens based on the research of Wang^8^. In addition, because the cross-shaped specimen can more accurately simulate the biaxial force state of the propellant, it has been widely used in recent years. Qiang H F^11^ carried out a biaxial tensile test of HTPB propellant based on the central thinning bathtub-shaped test piece through a biaxial testing machine; Jia Y G^12^ also calculated a square thinning cross-shaped test based on ANASYS simulation and carried out a biaxial tensile test of composite solid propellant; Jalocha^13^ believed that the method of grooving the wall of the specimen and thinning in the central area could not effectively characterize the biaxial properties of the propellant. For this purpose, a biaxial tensile test of composite solid propellant was carried out using a non-slotted test piece with an arc transition on the wall. However, the above test methods can only achieve biaxial tension with a single loading ratio, and can’t fully simulate the complex stress state of the engine at the moment of ignition. Therefore, a variable ratio biaxial tensile test method needs to be developed. In addition, the macroscopic mechanical properties of propellants are often closely related to the mesoscopic structure. Numerical simulation methods are widely used in the mesoscopic damage analysis of solid propellants due to their high efficiency and low cost. The establishment of mesoscopic simulation models mostly relies on high-precision observation experiments and random filling algorithms. The commonly used observation methods include Optical Microscope (OM)^14^, Scanning Electron Microscope (SEM)^15,16^ and Computed Tomography (CT)^17,18^. The key to numerical simulation calculation lies in the acquisition of material parameters, in which the mechanical properties parameters of the propellant matrix and particles can be obtained through experiments, while the parameters between the interfaces need to be introduced into the cohesive force model^19,20^. At present, researchers have carried out a large number of mesoscopic simulation studies under uniaxial conditions ^21–24^, but the research on propellants under biaxial conditions is not deep enough. Therefore, in order to study the damage evolution process of propellant under real loading conditions and explore its meso-damage mechanism, it is necessary to carry out the simulation calculation of solid propellant under biaxial loading conditions.

In this paper, aiming at the actual loading state of the propellant during the service process of the solid rocket motor, a variable ratio biaxial tensile mechanical property test of solid propellant was carried out through the self-designed new test fixture and biaxial tensile test piece.Then the initial morphology of the HTPB composite solid propellant was scanned and reconstructed by precision Micro-CT, and a clear two-dimensional mesoscopic structure inside the solid propellant was obtained. The number, size and area ratio of AP particles in the reconstructed image were counted. Based on the analysis results, a two-dimensional mesoscopic numerical model of the propellant was established by using the random filling algorithm, and the damage evolution of the propellant under the biaxial tensile load was numerically simulated based on the bilinear cohesion model. The purpose is to obtain the real mechanical response of the propellant in the actual working process through the mechanical property test, and establish the input and output of the mechanical response through the given loading conditions, so as to evaluate whether the propellant grain fails under the given conditions, which determines whether the solid rocket motor can be used normally. The numerical simulation can reveal the load transfer process between the meso components and the damage evolution law of the propellant, and explain the reason for the change of the macro mechanical response to a certain extent. Together, they provide reference for SRM structural integrity assessment.

## Test methods

### Test materials and test pieces

This paper selects HTPB composite solid propellant as the research object, and its components mainly include binder matrix, oxidant AP particles, metal fuel Al particles and other additives, the specific formula ratio is shown in Table 1. In addition, in order to reduce the influence of the production process on the test results, this paper selects the test pieces produced in the same batch to carry out the performance test.

**Table 1:** HTPB propellant formulation.

Propellant	Component	Mass fraction (%)
HTPB	HTPB (Hydroxyl terminated polybutadiene)	6.0–7.0
	AP (Ammonium perchlorate)	69.5
	AL (Aluminum powder)	18.5
	DOS (Di-2-ethylhexyl sebacate)	3.4
	MAPO (Tri-(2-methy-1-aziridinyl) phosphine oxide)	0.05–0.10
	TDI (2,4-Toluene diisocyanate)	1.0–2.0
	Additives (liquid)	0.5–1.0

In order to realize the variable-scale biaxial tensile test, the configuration of the test piece should meet the following requirements: (1) The configuration is relatively simple, the production process is less complicated, and it is easy to carry out biaxial tensile mechanical properties test after being assembled with the test fixture and testing machine; (2) After being loaded, deformation occurs in both directions at the same time, the stress in the central area are evenly distributed, and the area with uniform distribution is large; (3) The stress concentration in the non-central area is low and the area is small, and the the stress of central area should be larger, that is, the fracture failure starts from the central region. Based on the above requirements, this paper adopts the cross-shaped test piece configuration, and proposes the following optimization indicators:

- (1) Stress concentration factor *α*: the ratio of the maximum stress $\sigma_{{{\text{max}}}}$ in the stress concentration area of the test piece to the mean value $\overline{\sigma }$. The smaller the value is, the less obvious the stress concentration phenomenon of the test piece is. The calculation expression is:
  1 $$ \alpha = \frac{{\sigma_{{{\text{max}}}} }}{{\overline{\sigma }}} $$
- (2) Plane stress dispersion *CVs*: used to characterize the uniformity of the stress level of the test piece in the test area. The lower the dispersion, the higher the uniformity, and the higher the reliability of the corresponding test results. The calculation expression is:
  2 $$ CV_{{\text{s}}} = \frac{{\sqrt {\sum\limits_{{{\text{i}} = 1}}^{N} {(\sigma_{{\text{i}}} - \overline{\sigma })/N} } }}{{\overline{\sigma }}} $$
  in this equation, $\sigma_{{\text{i}}}$ is the stress of a single element, $\overline{\sigma }$ is the average stress in the test area, and *N* is the number of elements.
- (3) The priority failure coefficient *β*: the ratio of the maximum stress $\sigma^{1}_{{{\text{max}}}}$ in the test area to the maximum stress $\sigma^{2}_{{{\text{max}}}}$ in the non-test area. The larger the value, the higher the priority of the test area failure when the test piece is subjected to biaxial tensile load, and the more reliable the experimental results are. The calculation expression for:
  3 $$ \beta = \frac{{\sigma^{1}_{{{\text{max}}}} }}{{\sigma^{2}_{{{\text{max}}}} }} $$

**Figure 1:**
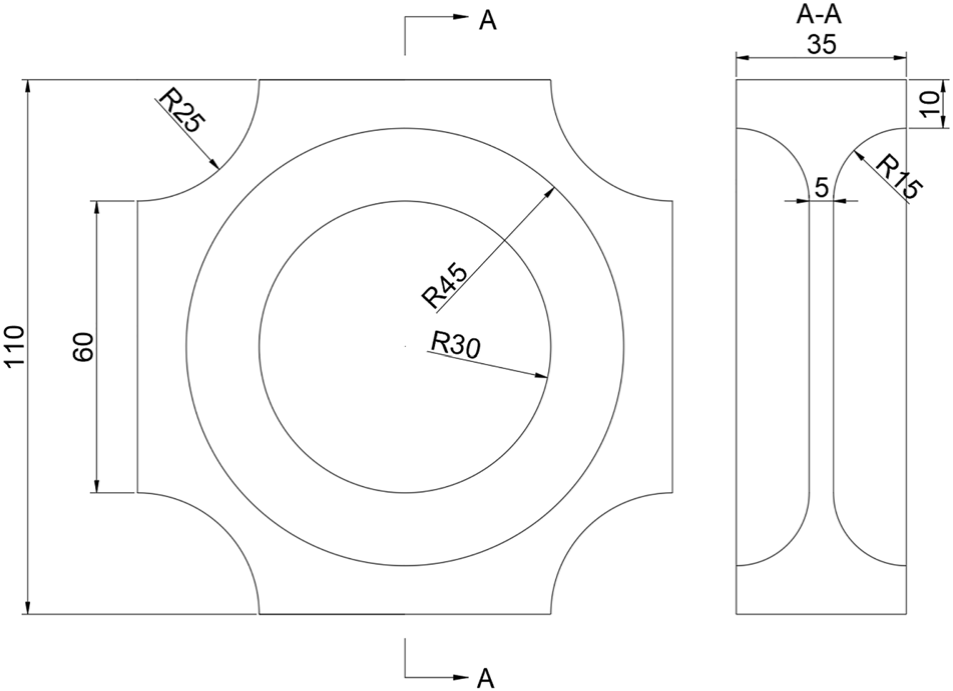
Dimensions of the test piece.

**Figure 2:**
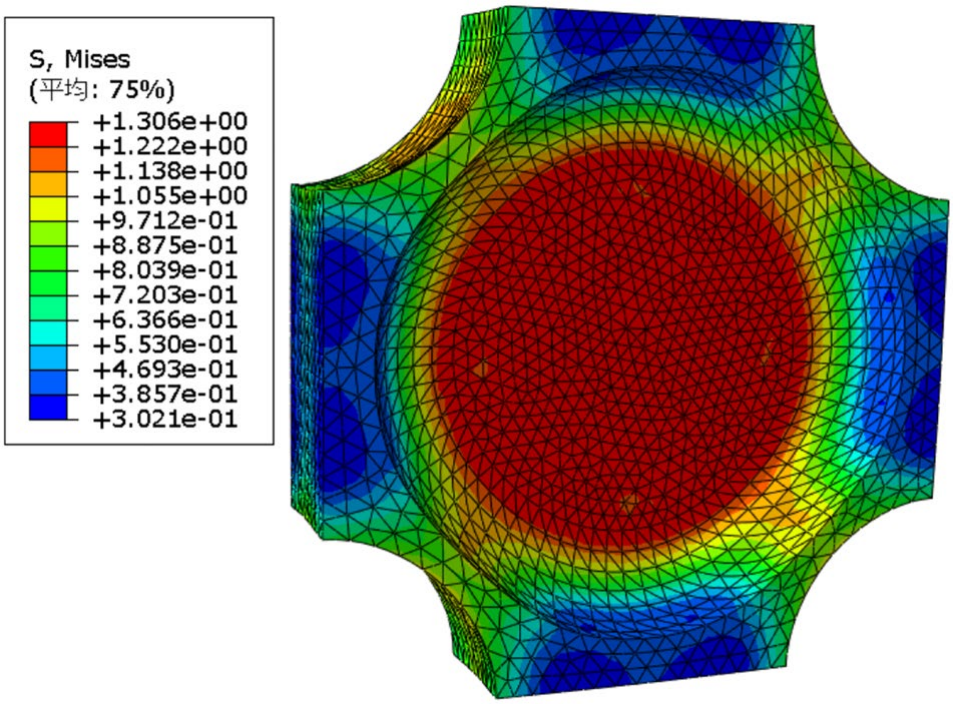
Mises stress of test piece Deformation.

Based on the above indicators and requirements, it is found that the configuration shown in Fig. 1 is the optimal configuration through the calculation and analysis of the deformation of the cross-shaped test pieces with different configurations on the ABAQUS software. That is, the center-thinning cross-shaped test piece with arc transition chamfering is used. Figure 2 shows the Mises stress diagram of the test piece under 20% strain under biaxial tensile load. In the calculation process, the propellant adopts the viscoelastic constitutive model in the form of Prony series^25^, the Poisson's ratio is 0.495, and the mesh is C3D8RH element. It can be seen from Fig. 2 that the maximum stress occurs in the central area , and the stress value in this area is generally larger than that in the non-central area, and the preferential failure coefficient *β* is 1.34, which is sufficient to ensure the test piece starts to break from the center area. In addition, the stress concentration coefficient *α* in the central area is close to 1, and the plane stress dispersion *CVs* is close to 0, that is, the stress concentration phenomenon of the test piece with this configuration is not obvious and the stress distribution uniformity is high, which meets the design requirements. After the test pieces were processed, they were placed in a drying oven for drying treatment.

**Figure 3:**
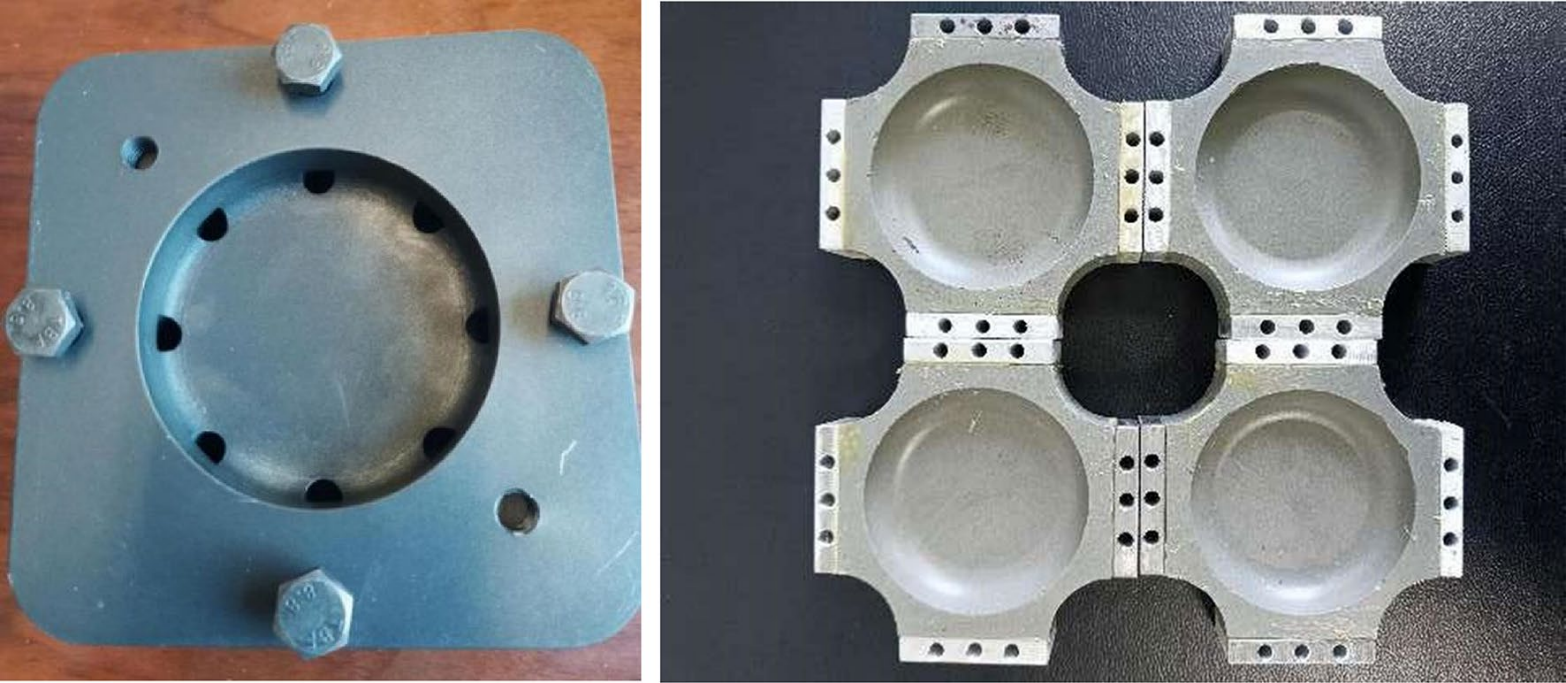
Propellant moulds and test pieces.

As there is no manufacturing standard for biaxial tensile test pieces at present, we listened to the opinions of industrial departments and adopted the casting production with the highest fault tolerance rate. First, uniformly mix the propellant formula shown in Table 1 in the environment of 58 °C in proportion. Then coat the mold release agent on the surface of the customized mold, and pour the mixed propellant slurry into the mold. Finally place the mold in the environment of 20 °C at a constant temperature, and take out the propellant sample after the slurry is completely solidified and cooled. Figure 3 shows the mold of the production test piece and the test piece after bonding the metal block. The purpose of bonding the metal block is to install the propellant test piece on the fixture. After processing, the test pieces shall be placed in the drying oven for drying.

### Test methods

**Figure 4:**
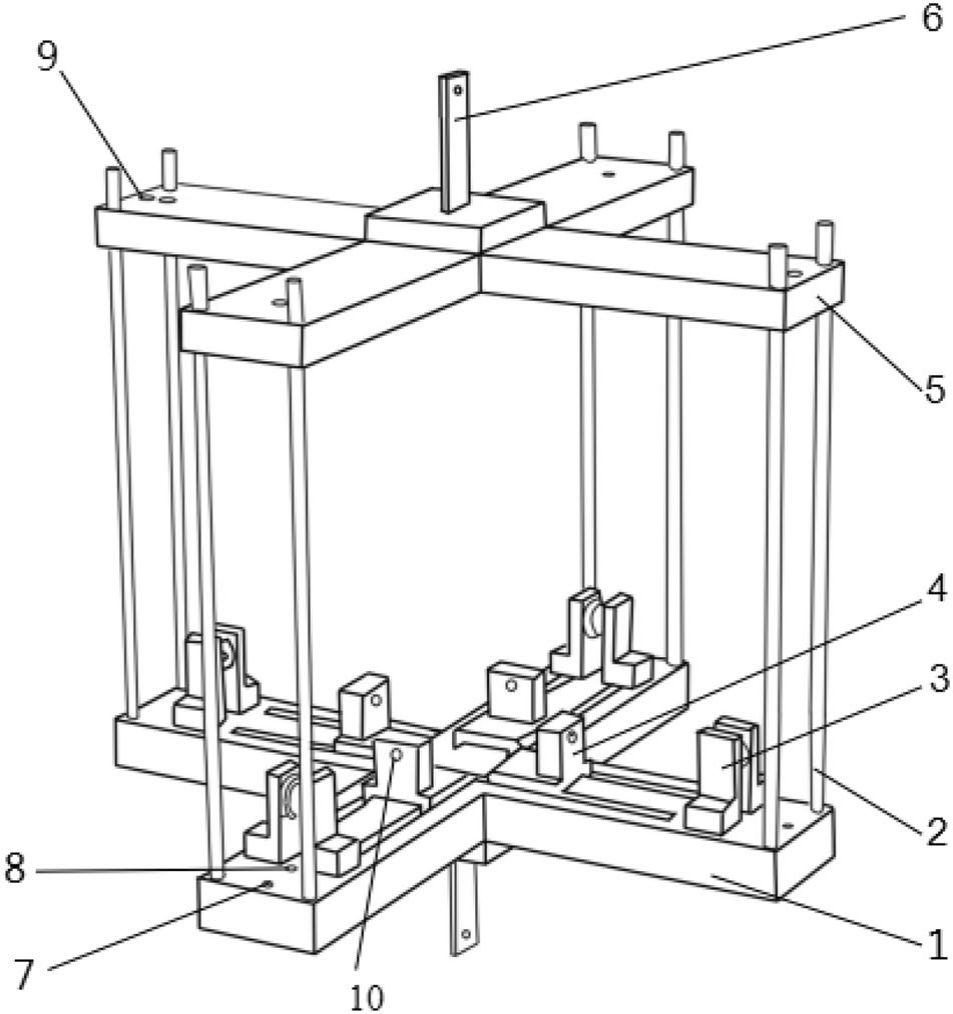
Test fixture (1-lower rail; 2-guide rod; 3-pulley; 4-slider; 5-upper rail; 6-clamp; 7-support rod connecting threaded hole; 8-lower rail preload threaded hole ;9- preload threaded hole on upper guide rail; 10-slider preloaded threaded hole).

**Figure 5:**
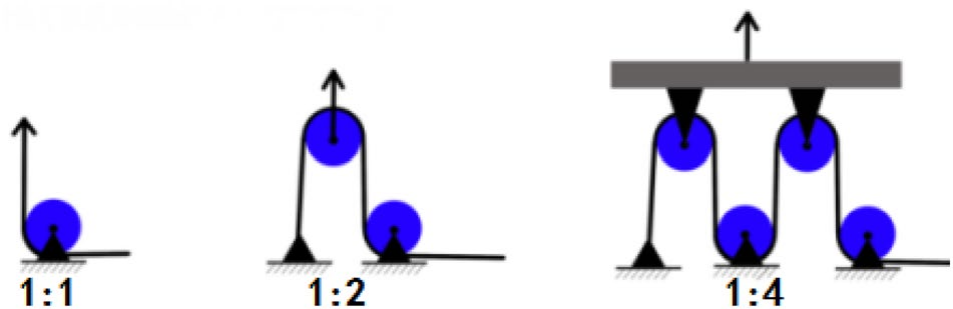
Variable proportion scheme based on pulley combination.

**Figure 6:**
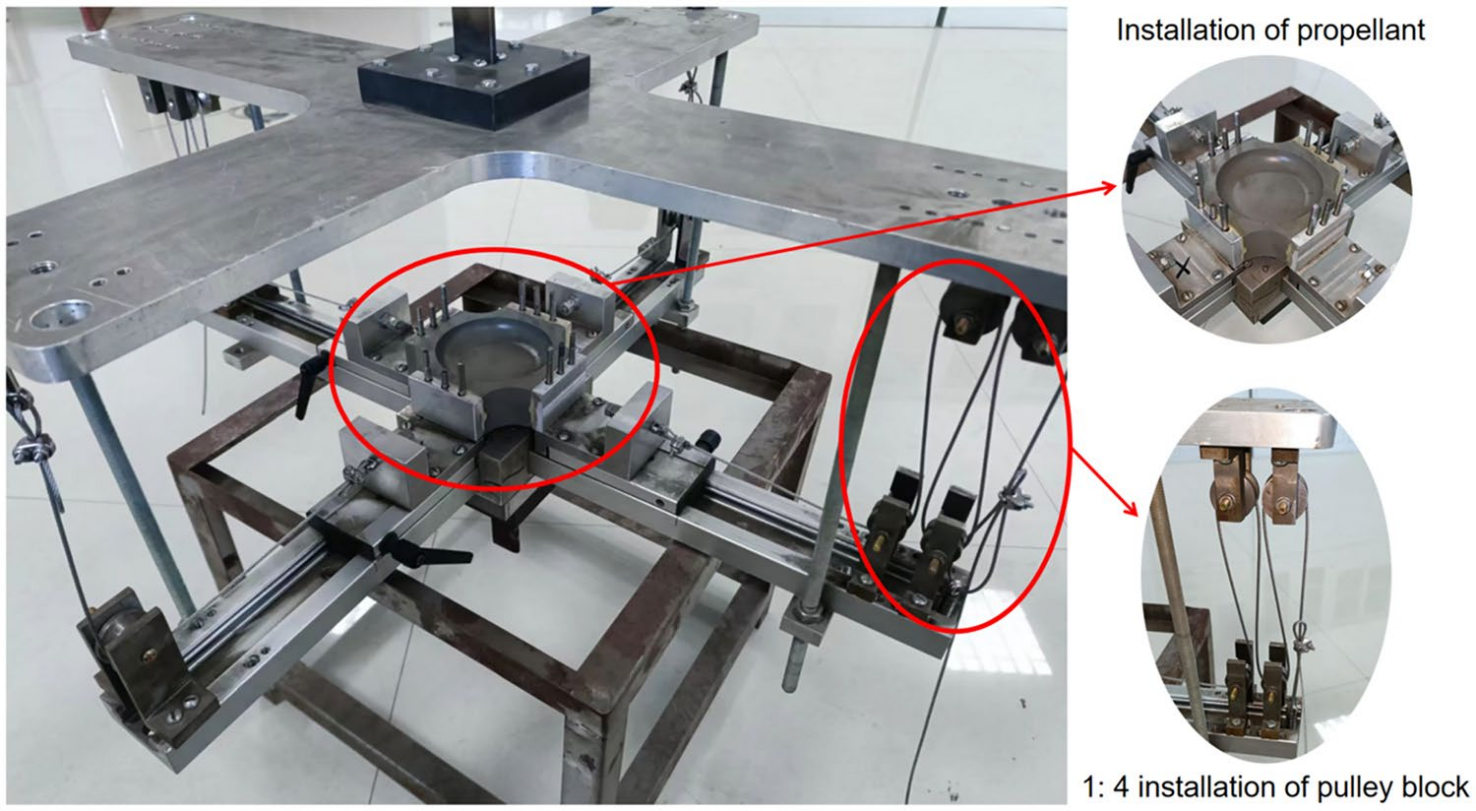
Assembly diagram of propellant test piece and test fixture under 1:4 loading.

Currently, the commonly used tensile testing machines for universal materials are generally unidirectionally driven and loaded, and can only achieve uniaxial tension, while the biaxial tensile testing machines are expensive and have certain limitations, and they tend to only be able to perform biaxial tensile tests at lower rates. Therefore, in order to improve the universality of this test piece and the convenience of carrying out higher strain rate tests later, this paper considers the advantages and disadvantages of the existing hinge type^26^, slider type^27^, lever type^28^ and pulley type^29^ biaxial stretching clamps, and a special biaxial tensile test fixture as shown in Fig. 4 is designed. Among them, the upper guide rail 5 and the lower guide rail 1 are connected by the guide rod 2, the lower guide rail 1 is provided with a groove, the slider 4 can slide freely on the groove, and the slider is composed of a connecting block and a sliding block. The solid propellant test piece is connected with the connecting block part by means of pins, and the preloaded threaded rod passes through the threaded through holes on the connecting block part and the sliding block part to fix it. The wire rope passes through the pre-tightening threaded hole 9 of the upper guide rail, the pulley 3 and the pre-tightening threaded hole 10 of the slider in turn, and is fixed at both ends of the parts 9 and 10 to realize the transmission of the tensile load. Using this fixture, the uniaxial tensile load acting on the upper and lower chucks can be converted into biaxial tensile loads of different proportions through different numbers of pulleys (shown in Fig. 5), so that the propellant test piece shown in Fig. 1 undergoes biaxial tensile deformation. Figure 6 is the physical diagram of the assembly of the test piece and the test fixture when biaxially stretched at a loading ratio of 1:4.

The test adopts the SZL-100 universal material tensile testing machine produced by Changchun Institute of Mechanical Sciences. The device is equipped with a force sensor and a displacement sensor in the loading direction, which can measure force and displacement synchronously in real time. The maximum tensile speed in one direction is 500 mm/min and can achieve a maximum load of 100 KN, which meets the conditions required for the test. Biaxial tensile mechanical properties tests were carried out on the testing machine at room temperature with different strain rates (0.01, 0.05, 0.2 s^−1^) and different loading ratios (1:1, 1:2, 1:4), the corresponding loading rate is shown in the Table 2. During the loading process of the test, the corresponding loading displacement and load value are recorded by the testing machine software. In order to improve the reliability of the test results, 5 groups of repeated experiments were performed under each experimental condition, and the results of the subsequent experiments were the mean of the 5 groups of data.

**Table 2:** Loading rate under different loading conditions.

Loading ratio	1:1	1:2	1:4
Loading rate (mm/min)	19.09 (0.01 s^−1^)	12.07 (0.01 s^−1^)	6.55 (0.01 s^−1^)
	95.46 (0.05 s^−1^)	60.37 (0.05 s^−1^)	32.74 (0.05 s^−1^)
	381.84 (0.2 s^−1^)	241.50 (0.2 s^−1^)	130.97 (0.2 s^−1^)

### Acquisition method of stress and strain

Unlike uniaxial tension, biaxial tension specimen has complex configuration and special stress state, so it is difficult to calculate its stress and strain directly. Therefore, referring to the method in references^13,30^, this paper uses the elastic small deformation finite element calculation to determine the transfer function between the stress/strain in the central area of the cross shaped specimen and the force/displacement at the wall end, so as to estimate the stress and strain in the central area of the specimen.

**Figure 7:**
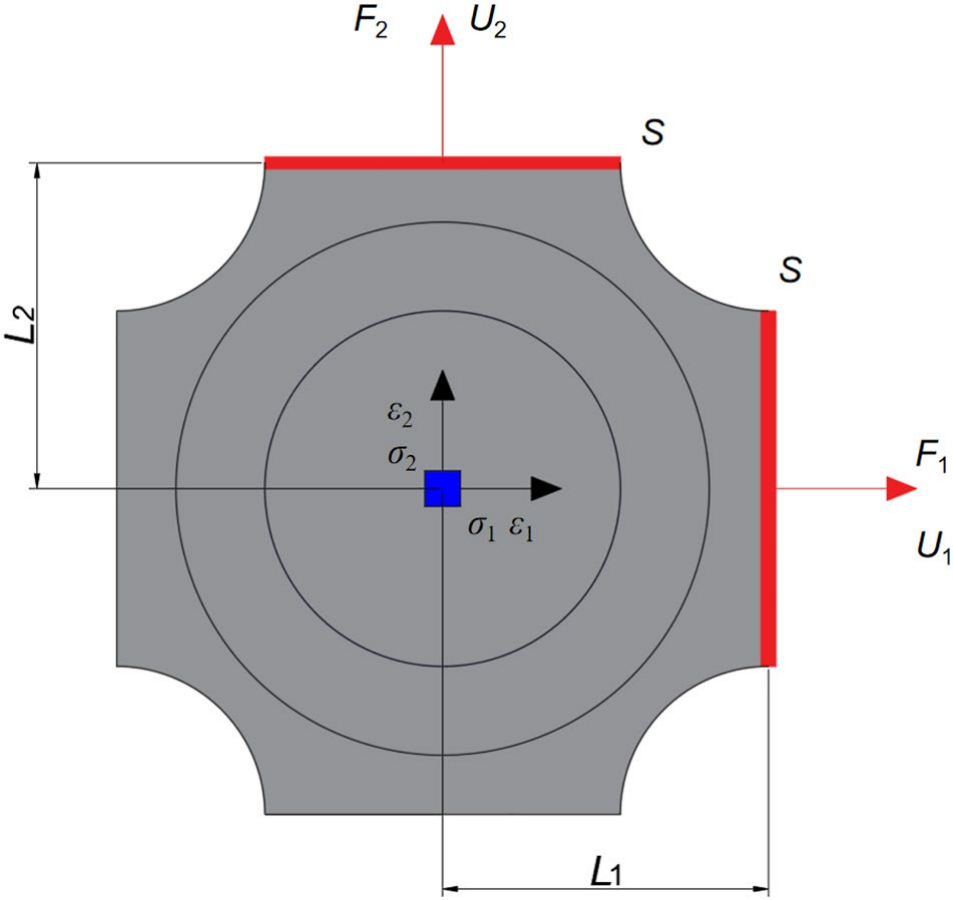
Schematic diagram of stress and strain calculation (*F*_i,_ *U*_i,_ *S* are the force, displacement and area of the wall end of the specimen respectively; *L*_i_ is the distance from the wall end to the center of the specimen; *σ*_i_ and *ε*_i_ are the stress and strain in the central area of the specimen respectively).

**Figure 8:**
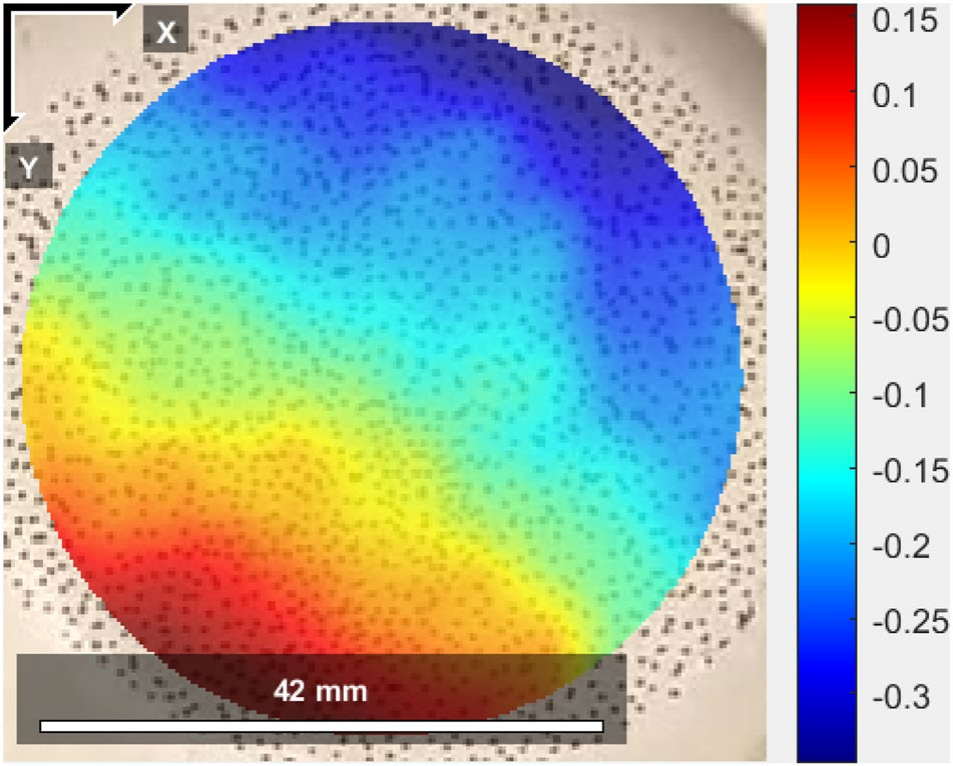
Strain nephogram obtained by DIC method.

Figure 7 is the schematic diagram of the stress and strain calculation of the test piece. Because the wall end is bonded to the metal block of the same shape, the area *S* of the wall end remains unchanged during the stretching process. *F*_i_ and *U*_i_ can be calculated by the sensor of the testing machine, and the stress and strain in the center of the test piece can be obtained by the following formula:

4 $$ \sigma_{{\text{i}}} = L_{\sigma } \frac{{F_{{\text{i}}} }}{S}\;\;\varepsilon_{{\text{i}}} = L_{\varepsilon } \frac{{U_{{\text{i}}} }}{{L_{{\text{i}}} }}\;\;\;({\text{i}} = {1},\;{2)} $$

where, *L*_σ_ and *L*_ε_ is the transfer function determined by the finite element solution, which are computed from strain and stress values in the center of the specimen and the values at the end section by minimizing the least squares distance. In addition, in order to verify the accuracy of this method, this paper uses Digital Image Correlation (DIC)^31,32^ to verify the deformation process of the specimen. First, spray uniform white paint on the surface of the propellant specimen, then make random speckle with black paint, and fix the propellant on the fixture after it is completely air dried; Secondly, a high-speed camera with a fixed position is used to capture the tensile process of the specimen, and the captured images are collected and processed frame by frame; Finally, Matlab program is used to obtain the strain field of the collected pictures. Figure 8 shows the strain nephogram obtained by DIC method during the deformation of propellant. After comparison, the deviation between the DIC method and the transfer function calculation result is less than 10%, which verifies the feasibility of the transfer function calculation method.

### Failure behavior of propellant

**Figure 9:**
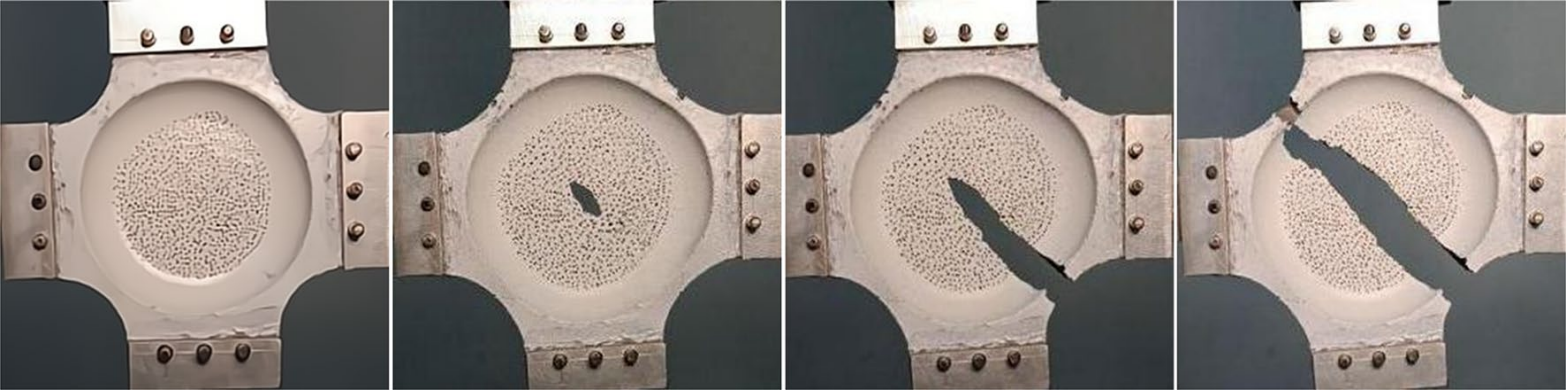
Failure process of propellant specimen.

By taking photos of the tensile failure process of the test piece under biaxial 1:1 loading, it is found that the circular area in the central area of the test piecegradually increases at the beginning of the tensile process, and when the test piece deforms to a certain extent, some small cracks gradually appear in the central area of the test piece.With the increase of tensile strain, the small cracks in the central area of the test piece gradually increase and form holes that connect together to form large cracks. Subsequently, these cracks continue to expand towards the corners of the limbs of the test piece until they run through the whole test piece, as shown in Fig. 9. The tensile load on the limbs of the test piece forms a resultant force in the central area of the test piece, that is, the tensile load on the adjacent ends combines a larger load pointing to the direction of the limb chamfer, so that the test piece breaks along the direction of the four limb chamfers. At the same time, the central area of the test piece is the thinnest part of the whole test piece, while the connection of the four limbs of the test piece is relatively thick, Therefore, the crack initiation of the test piece occurs in the relatively central area of the test piece. The test phenomenon is consistent with the test expectation, that is, the first place where the test piece is damaged is the central effective area of the test piece, which meets the requirements for the design and preparation of the cross shaped test piece.

In addition, according to the configuration of the test piece and the loading method, it is easy to know that the closer the central area of the test piece is, the smaller the strain will be. However, the propellant is destroyed and failed from the center in Fig. 9, which indicates that when the propellant is subjected to biaxial tensile load, it often fails because it reaches the maximum tensile strength, rather than the maximum strain. The subsequent biaxial tensile tests with different loading ratios also verified this point.

## Test results and discussion

### Stress–strain curve characteristics

**Figure 10:**
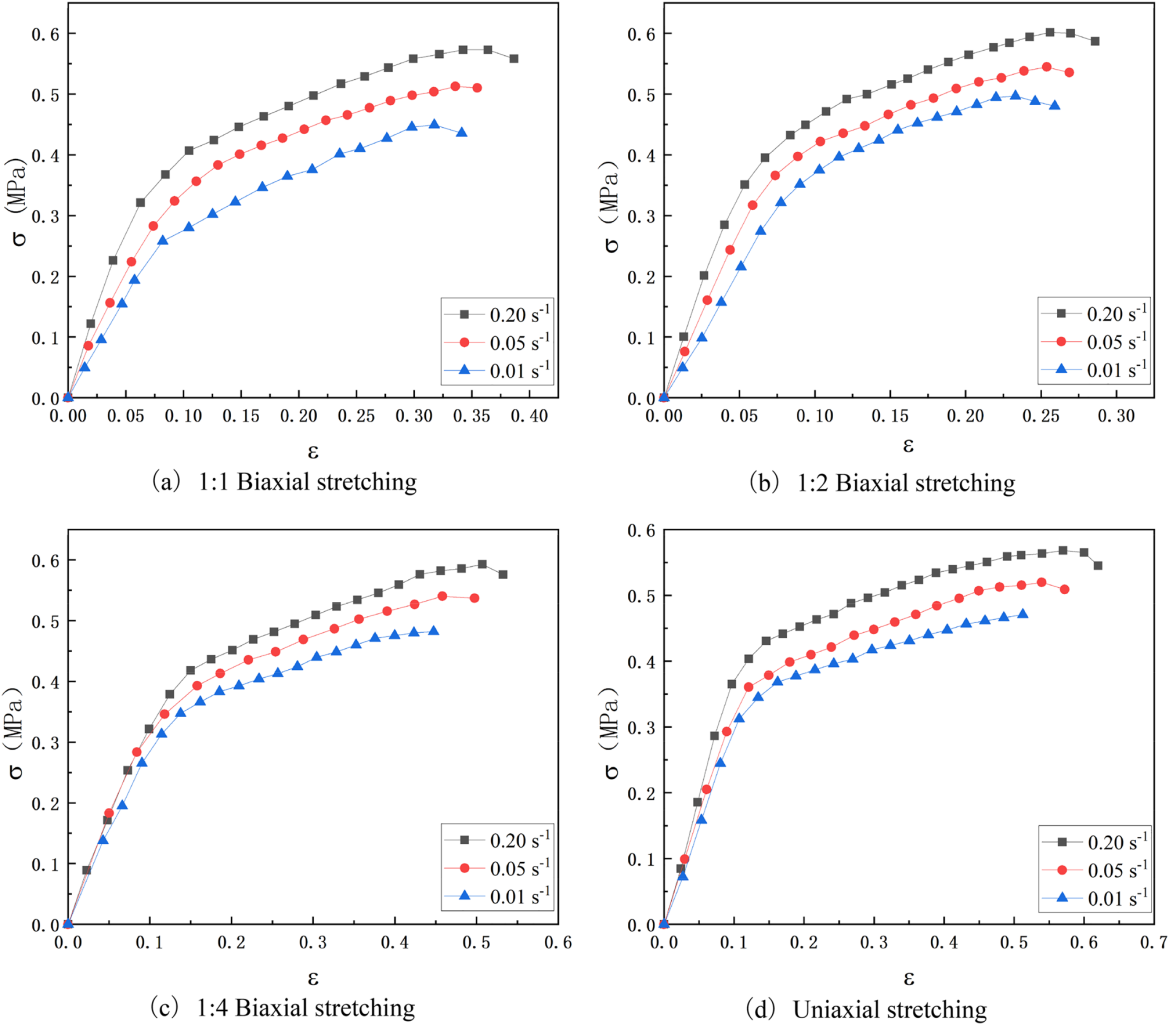
Tensile stress–strain curves of HTPB propellant at different strain rates and different stress states at room temperature.

Figure 10 is the stress–strain curve of the HTPB propellant obtained under the test conditions. For comparison, the uniaxial tensile stress–strain curve of the propellant under the corresponding loading temperature and strain rate is also given, as shown in Fig. 10(d). It can be seen from Fig. 10 that the stress–strain of HTPB propellant has the following characteristics under variable-ratio biaxial tensile loading.

- 1. Biaxial stretching and uniaxial stretching curves have similar characteristics in terms of curve characteristics, and both show a typical three-segment curve. The initial linear segment appears first, at this time, the propellant has not been damaged at the beginning of the load, showing the properties of a linear elastic material, that is, the stress changes linearly with the strain; Then there is a damage evolution period, at this time, the propellant begins to be damaged and the damage starts to accumulate with the increase of strain, which shows that the change of stress tends to be gentle with the increase of strain.; Finally, there is a failure section, the propellant begins to have obvious cracks, the cracks continue to expand until they break as the strain increases, and the stress also begins to decrease.
- 2. Under the same loading conditions, the maximum elongation of propellant under biaxial tension is significantly lower than that under uniaxial tension, which is generally 45–85% of that under uniaxial tension, and is significantly affected by stress state and loading rate. The maximum elongation under 1:2 biaxial tension loading is only 45% of that under uniaxial tension, which is similar to the research results obtained by Wang through strip test^8,33^ This is mainly due to the asymmetry of two-way loading, which is very easy to cause large deformation in some parts and failure, thus leading to the most obvious drop in the overall elongation of the propellant. However, under the 1:4 biaxial tensile loading, the elongation of the propellant has obviously rebounded, which is due to the large difference between the loading ratios of the two directions, which results in that the main stretching direction is not obviously constrained by the other direction, and the macromolecular chain is easy to slip along a certain direction. Therefore, it is not difficult to speculate that with the increasing proportion of biaxial tension loading in both directions, its elongation will continue to approach to uniaxial tension. In addition, with the increase of loading rate, the maximum elongation of propellant under different stress states will also increase. This may be because the higher loading rate makes the damage degree of propellant less when it is damaged, and the degree of dehydration of internal particles is less, which leads to better ductility of propellant^34^.
- 3. Under the same loading conditions, the maximum tensile strength of the propellant under biaxial tension is slightly higher than that under uniaxial tension, and its ratio is between 1.0 and 1.1. This indicates that biaxial tension has a strengthening effect on the strength of the propellant, which is similar to the results of the biaxial tensile strength research carried out by Wang and Zhang Lihua^8,35^. It is not difficult to explain this rule from the perspective of macromolecular mechanics: the macromolecular chains in the propellant polymer are constrained in two directions under biaxial tensile load, unlike the slip between the molecular chains along one direction when under uniaxial load, which leads to lower elongation of the propellant under biaxial load, and requires higher stress to damage. In addition, similar to the maximum elongation, the maximum tensile strength of the propellant also increases with the increase of the strain rate, which can also be explained by the conclusion in literature^34^. However, the strength and regularity of propellant under different stress states do not change obviously, which needs to be further discussed in future research.

### Characteristics of dewetting point

**Figure 11:**
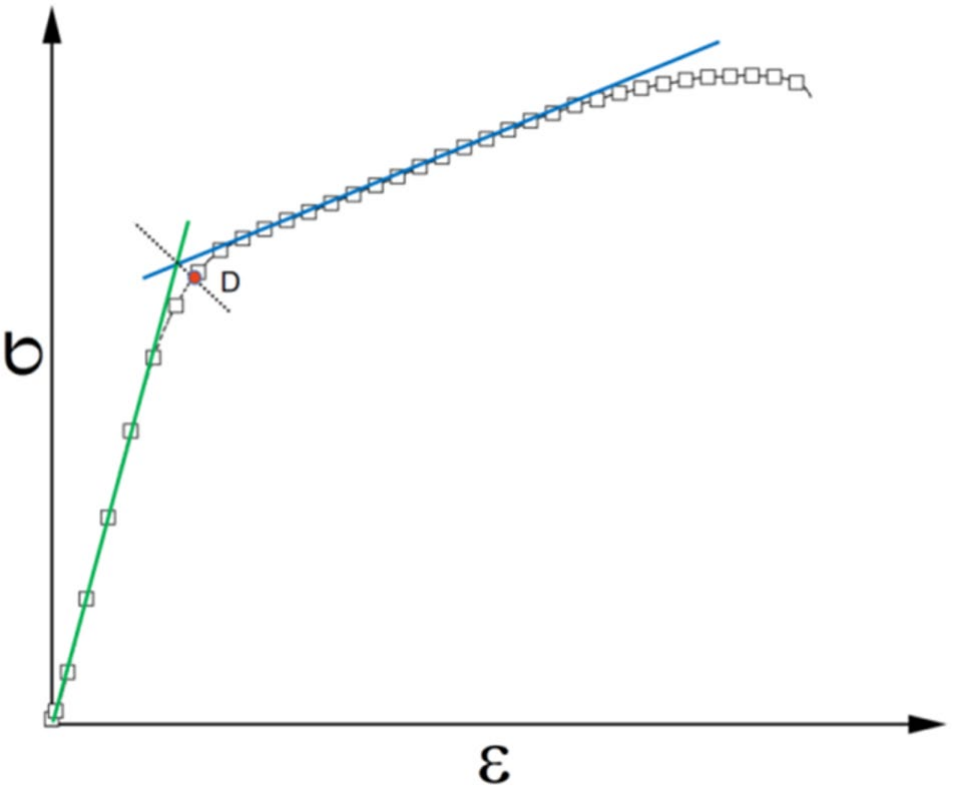
Determination method of dewetting point.

**Figure 12:**
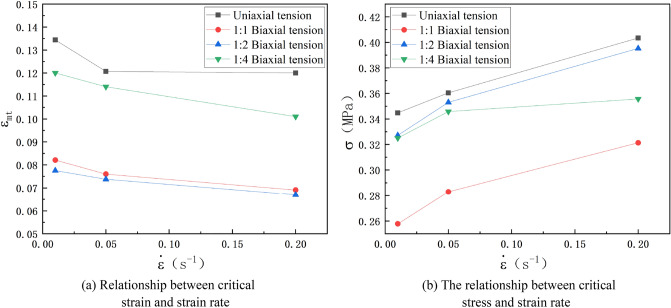
Variation curves of critical stress and strain of HTPB propellant at dewetting point under different loading conditions at room temperature.

As a high-energy viscoelastic composite material, composite solid propellant needs to be filled with high volume fraction of solid particles for achieving good ballistic mechanical properties and storage performance. However, the interface between the solid particles and the matrix is often the weak point of the propellant's carrying capacity. A study^36^ pointed out that the strength between the two-phase interface largely determines the mechanical properties of the propellant. The nonlinearity of solid propellant deformation comes from the interface failure (ie dewetting) between the solid filling particles inside and the matrix^37^, and the critical point of the stress–strain curve is defined as the dewetting point. Reference^38^ in this paper defines the dewetting point of HTPB propellant under different loading conditions directly from the stress–strain curve based on the method shown in Fig. 11. The critical stress and strain results at the dewetting point are shown in Fig. 12.

It can be seen from Fig. 12 that the variation law of the elongation at the dewetting point under different loading conditions is similar to the maximum elongation. The elongation at the dewetting point is the highest during uniaxial stretching, followed by 1:4 biaxial tensile loading, and finally as 1:2 biaxial tensile loading, which indicates that the propellant is more prone to dewet under biaxial tensile loading than uniaxial tensile loading. As the strain rate increases, the elongation at the initial dewetting point of the propellant decreases, but the strength increases, the dewetting point moves forward and upward with increasing strain rate, dewetting is more likely to occur. This is mainly because when reaching the same deformation, the propellant will be subjected to greater stress under high strain rate loading, so that the interface between the particles and the matrix is more likely to reach the critical strength and be damaged, and the rate of dewetting is also faster, which further promotes the occurrence of dewetting.

The variation law of the critical stress and strain at the above dehumidification point is closely related to the damage evolution of the mesostructure of HTPB propellant under different loading conditions. However, it is difficult to carry out the observation test of the damage evolution of solid propellant under biaxial loading at present. Therefore, In this paper, the relevant discussion and analysis are carried out through the meso-simulation calculation.

## Numerical calculation of meso-damage finite element

### Meso-structure model

**Figure 13:**
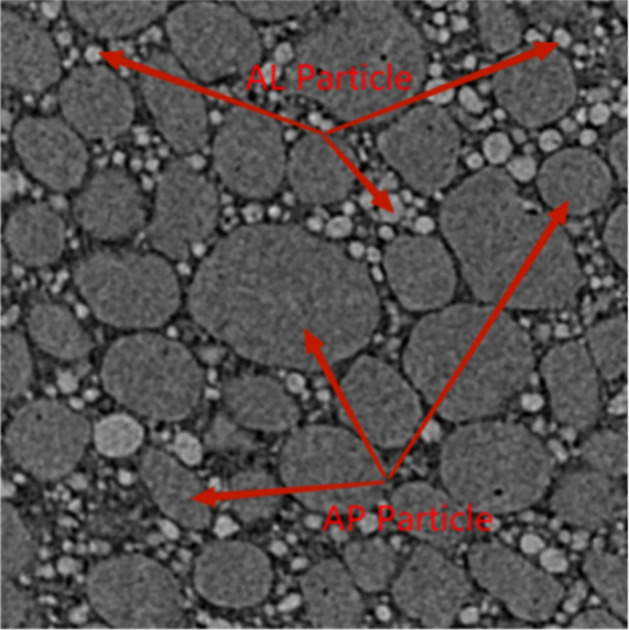
Real mesomorphology of HTPB propellant.

In order to establish a more realistic propellant meso-model, it is first necessary to obtain the real propellant meso-structure. In this paper, the HTPB propellant was scanned by the Skyscan 1172 Micro-CT equipment, and an area of 1000*1000 μm^2^ was selected for reconstruction. The reconstruction results are shown in Fig. 13. Since substances with different densities have different ability to absorb X-rays, different grayscale values will be shown in the reconstructed image. The denser the structure, the stronger the absorption of X-rays, resulting in a larger gray value and brighter in the reconstructed image. Therefore, the mesoscopic components of the HTPB propellant can be easily distinguished from the figure. Therefore, the mesoscopic components of the HTPB propellant can be easily distinguished from the figure: the brightest structure is Al particles, followed by AP particles, and finally the matrix. It can be seen from the figure that the shape of AP particles is mostly close to a circle or an ellipse, high-density filling in the matrix, and has a large size span. But the number of large-sized AP particles is small, the shape of the Al particles is approximately circular, and its size is much smaller than that of the AP particles, and is filled between the AP particles.

Secondly, it is necessary to use image processing methods to analyze the propellant mesostructure in the reconstructed area, which mainly includes the size, number and area percentage of AP particles. The idea of using the image processing method to analyze the reconstructed HTPB propellant mesomorphology is as follows: segment the area represented by the AP particles according to the different gray values, and then set a reference scale for the image to determine the size , quantity and area ratio of AP particle. According to the analysis, the number of AP particles in the selected area is 44, the area percentage of all AP particles is 53%. The size of AP particles is mostly between 100 μm and 200 μm, but there are also a small number of AP particles with larger sizes, whose size is greater than 300 μm.

**Figure 14:**
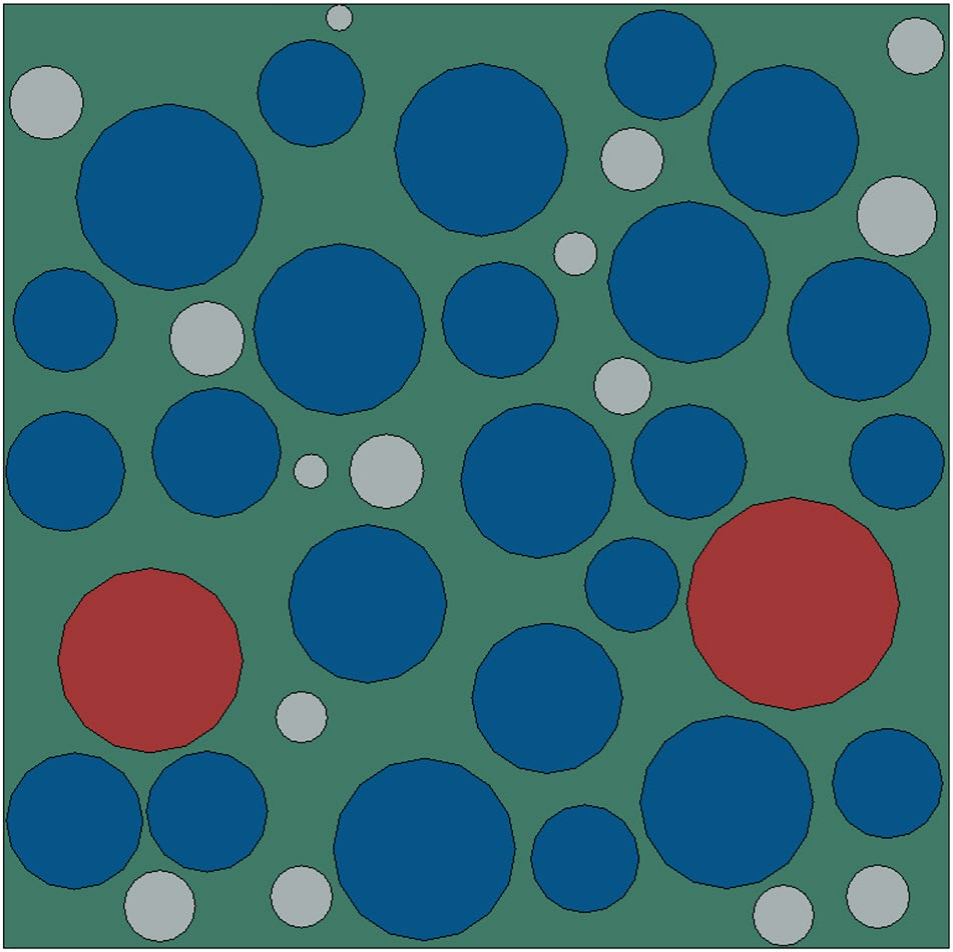
Meso-scale filled geometric model diagram.

Reference^39^ shows that the bonding strength between solid filler particles and the matrix is inversely proportional to the particle size. Since the particle size of Al particles is much smaller than that of AP particles, usually dewetting occurs only at the bonding interface between AP particles and the substrate. For this reason, the Mori–Tanaka method was used to equivalence Al particles into the matrix material when performing mesoscopic finite element calculations. Then, based on the AP particle gradation and area ratio statistically obtained in Table 3, a random filling algorithm was used to generate the meso-particle filling model of HTPB propellant as shown in Fig. 14. Among them, the size of the representative volume unit is the same as the size of the observation area selected in the experiment, that is, 1000*1000 μm^2^. Different colors in the figure represent AP particles of different particle sizes, and the area ratio of AP particles is 53%. The comparison between the grading of AP particles in the built model and the actual statistical value is shown in Table 3. Through the comparison, it can be found that the built model is in good agreement with the actual mesoscopic structure of the propellant.

**Table 3:** Statistical particle grading and modeled particle grading.

Particle size (μm)	Slice image geometric information		Generate geometric information of numerical model	
	Area proportion	Quantity	Area proportion	Quantity
200–350	0.07	2	0.07	2
100–200	0.41	29	0.41	24
25–100	0.05	13	0.05	15

### Material model and model parameters

**Figure 15:**
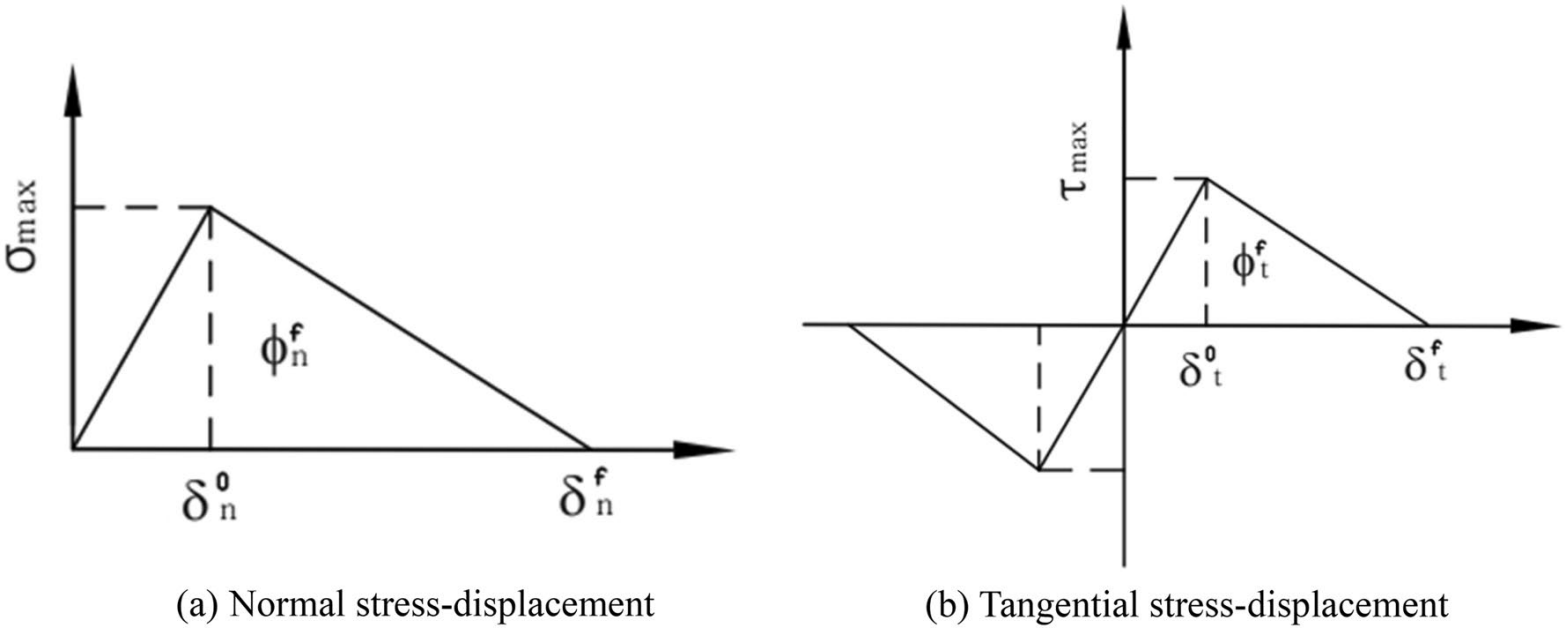
Cohesion model.

Cohesive Zone Model (CZM) was first used to study the fracture problem of brittle materials^20,40^. The basic idea is to regard the interface in the material as a bonding unit with a certain bonding strength, which describes the mechanical response of the interface by defining the relationship between the traction force and the displacement on the element. Due to its simple form, the bilinear cohesion model has been widely used in the numerical simulation of the mechanical behavior of the bonding interface. The typical bilinear cohesion model is shown in Fig. 15.

The bilinear cohesion model theory holds that the interface will not be damaged within a small deformation range, and the interface stress has a linear relationship with the opening displacement. When the opening displacement of the interface gradually increases to a critical value $\delta^{0}$, the interface stress reaches the maximum value, and damage occurs at the interface, and then the interface stress decreases with the increase of the opening displacement until it reaches the maximum opening displacement $\delta^{f}$ to invalid . The traction–separation law satisfied by the bilinear cohesion model is expressed as:

4 $$ T = \left\{ {\begin{array}{*{20}c} \sigma \\ \tau \\ \end{array} } \right\} = \left[ {\begin{array}{*{20}c} {\left( {1 - D} \right)k_{n} } & 0 \\ 0 & {(1 - D)k_{t} } \\ \end{array} } \right]\left[ {\begin{array}{*{20}c} {\delta_{n} } \\ {\delta_{t} } \\ \end{array} } \right] $$

where $\sigma$ and $\tau$ are the normal and tangential stresses of the interface, respectively; *k*_*n*_ and *k*_*t*_ are the normal and tangential stiffness of the interface, respectively; $\delta_{n}$ and $\delta_{t}$ are the normal and tangential opening displacements of the interface, respectively. *D* is the damage variable, which is used to judge whether the interface is damaged. When *D* < 0, there is no damage on the interface, when 0 < *D* < 1, the interface is damaged, when *D* = 1*,* the damage value of the interface reaches the maximum value and fracture occurs, *D* can be defined by the following expression:

5 $$ D = \left\{ {\begin{array}{*{20}l} 0 & {\left( {\delta \le \delta^{0} } \right)} \\ {\frac{{\delta^{f} \left( {\delta - \delta^{0} } \right)}}{{\delta \left( {\delta^{f} - \delta^{0} } \right)}}} & {\left( {\delta > \delta^{0} } \right)} \\ \end{array} } \right. $$

**Figure 16:**
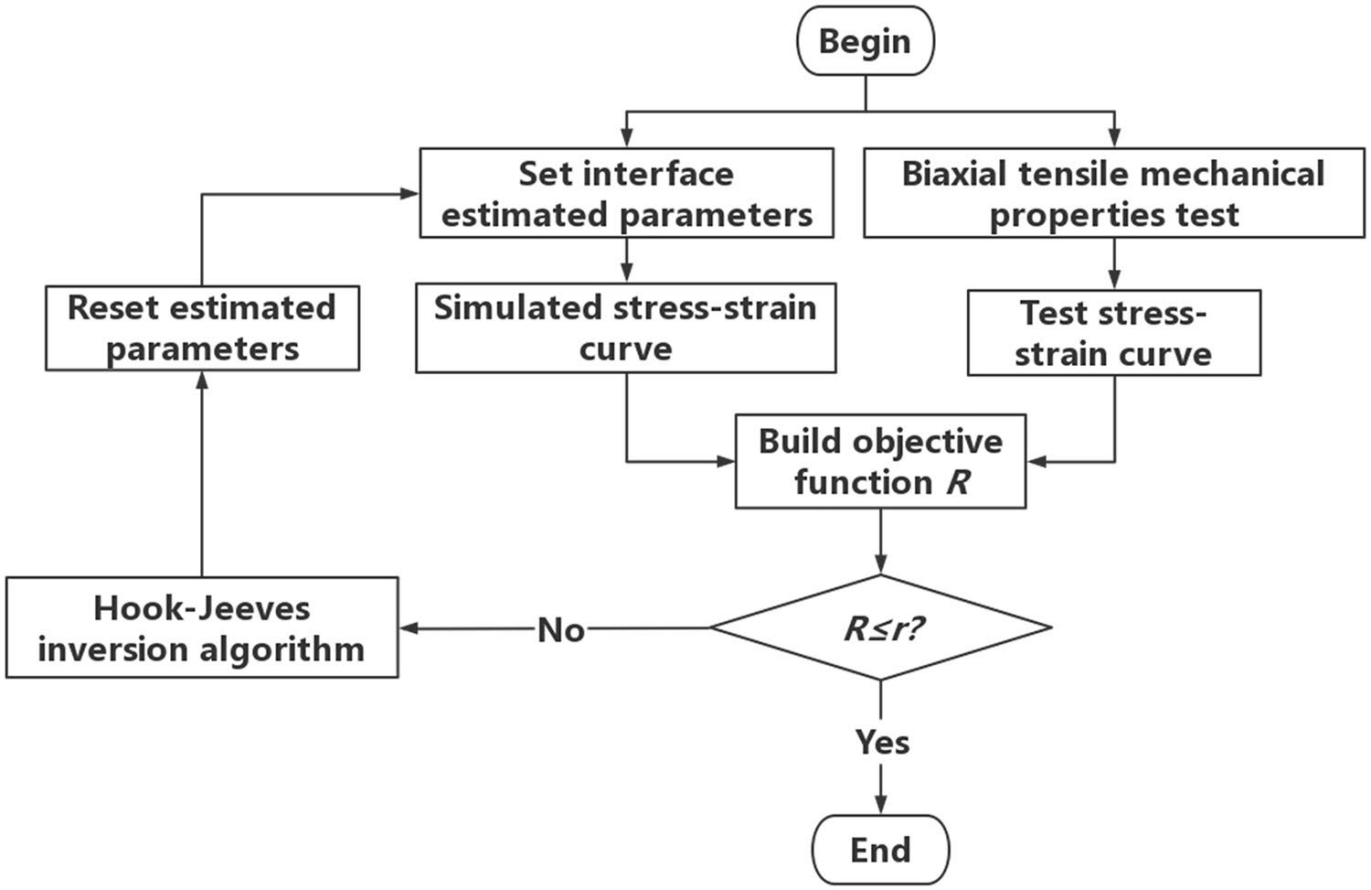
Inversion program of the paramete of CZM.

Since the modulus of AP particles is much larger than that of the matrix, the elastic constitutive model is used to describe the deformation characteristics of AP particles. The measured modulus of AP particles is 32,500 MPa, and the Poisson's ratio is 0.143. Secondly, the matrix material usually exhibits hyperelastic properties. Therefore, the reference^41^ uses the Ogden-Based hyperelastic model for description, and the model parameters are shown in Table 4. Finally, in order to reflect the interface failure between the solid filler particles and the matrix and the tear failure of the matrix during tensile loading, by writing a Python script program, the zero-thickness cohesion elements are inserted between the interface and the matrix element in this paper, and the bilinear cohesion model is selected. In this paper, the initial stiffness, bond strength and failure displacement of the cohesive force model are determined by inversion based on the Hook-Jeeves method^42^. The specific step flow is shown in Fig. 16. First, set the estimated bonding interface model parameter, and use this parameter for simulation calculation to obtain a set of simulation stress strain curves. Then, compare the test stress strain curve with the simulation stress strain curve and construct the objective function R \* MERGEFORMAT ^43^. Finally, set the error limit r, if the target function *R* > *r*, use Hook-Jeeves inversion algorithm to reset the new prediction parameters until the target function *R* ≤ *r*.

**Table 4:** Ogden model parameters of matrix.

Ogden model	*i*	*μ*_*i*_	*α*_*i*_
*N* = 3	1	0.44355	3.19509
	2	− 0.32115	3.91493
	3	0.09432	− 9.7668

In the calculation process, the secondary nominal stress and the failure displacement are used as the criteria for the damage initiation and evolution of the interface bonding element. In this paper, two cohesive force models are used in the simulation calculation, one is the fracture model of the bonding interface between AP particles and the matrix, and the other is the fracture model of the bonding interface between adjacent matrix elements, both of which are obtained through Hook-Jeeves inversion algorithm. The specific parameters are shown in the Table 5 .

**Table 5:** Mechanical parameters of interface.

Interface	Initial stiffness (Mpa/mm)	Bond strength (Mpa)	Failure displacement (mm)
Particle/matrix interface	1500	0.2	0.02
Matrix element interface	500	0.3	0.035

**Figure 17:**
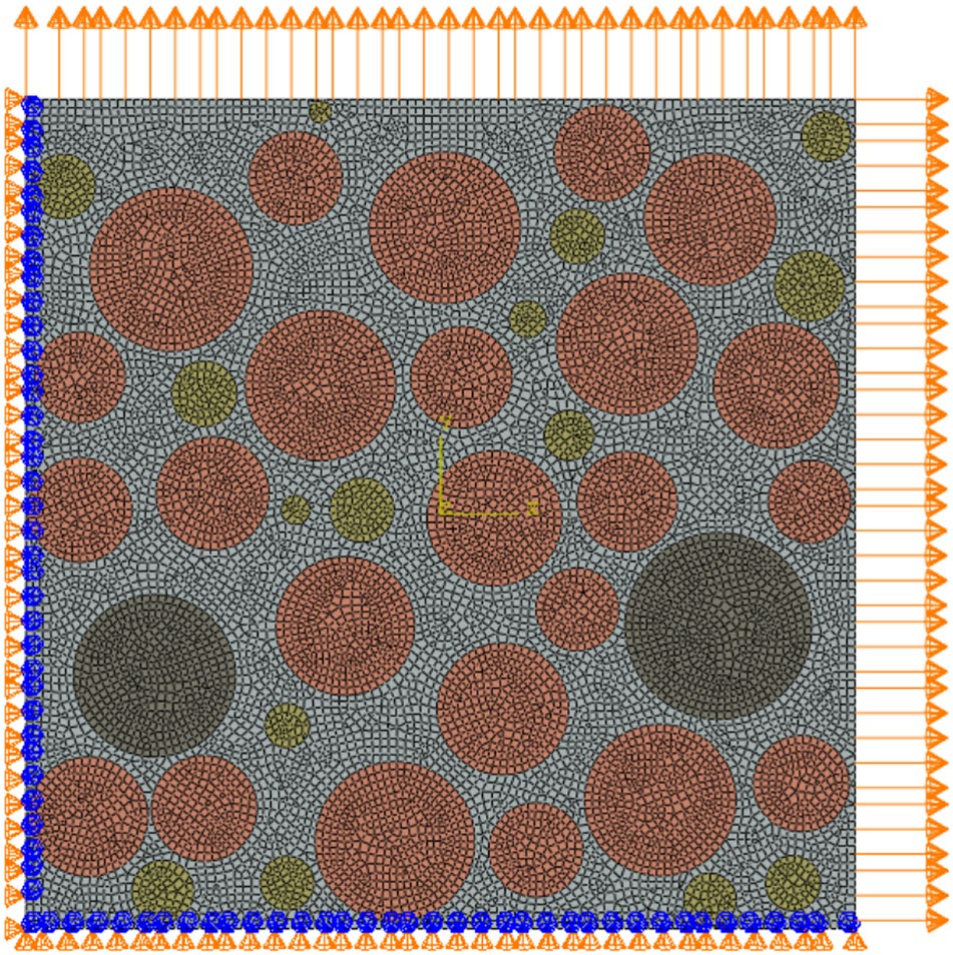
Meshing and boundary conditions of biaxial stretching.

The AP particle mesh adopts the four-node plane strain element CPE4. For the propellant matrix material, because its Poisson's ratio is very high, the four-node plane strain hybrid element CPE4H is used to mesh it. The four-node plane bonding element COH2D4 is used as the Cohensive element type to characterize the tearing of the AP particle/matrix interface and the matrix. In addition, a penalty function contact constraint needs to be defined at the interface to prevent mutual intrusion between elements. In order to simulate the biaxial tensile load, the normal displacement load is applied to the nodes on the adjacent two sides at the same time, and the normal displacement and rotation constraints are applied to the nodes on the other two sides. The application of boundary conditions is shown in Fig. 17. The simulation of uniaxial tensile load only needs to cancel the lateral displacement load on the basis of the former.

### Calculation results and discussion

**Figure 18:**
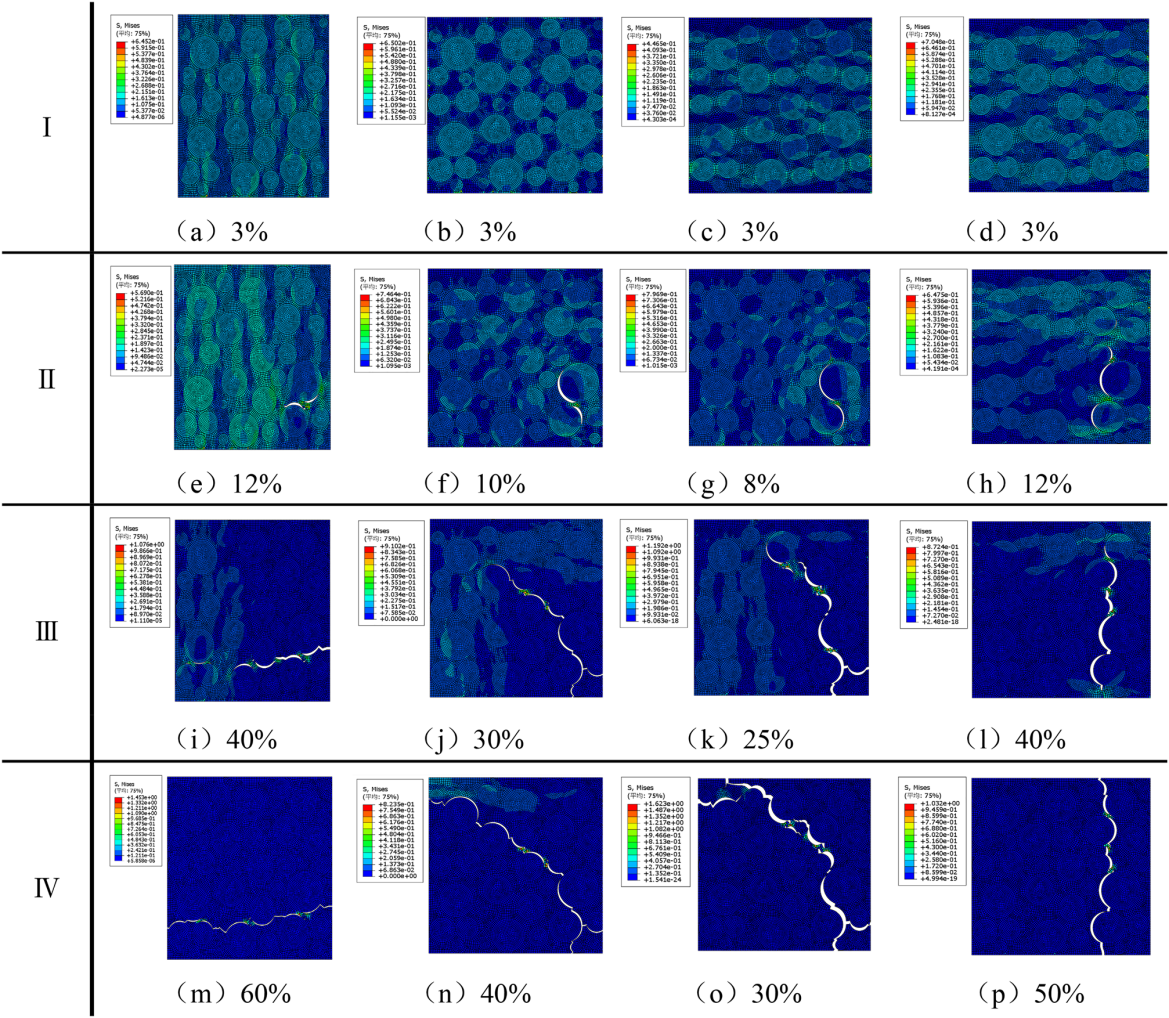
Stress nephograms of damage evolution under different loading conditions under 0.05 s^−1^ strain rate loading (I-Initial linear stage, II-Start-dewetting point, III-Damage evolution stage, IV-Fracture failure stage; a-Uniaxial tension, b-1:1 Biaxial tension, c-1:2 Biaxial tension, d-1: 4 Biaxial stretching).

In order to compare the damage evolution process of propellants under different stress states, the calculation results under different loading conditions under 0.05 s^−1^ strain rate loading are selected for analysis, and the stress nephogram is shown in Fig. 18. It can be seen from the calculation results that the fracture process of the propellant can also be divided into three stages, corresponding to the initial linear stage, the damage evolution stage and the fracture failure stage of the stress–strain curve, respectively. In the initial stage of stretching, due to the huge difference in stiffness between the AP particles and the matrix, the internal stress distribution of the propellant is extremely uneven, and the AP particles carry most of the load. At this time, the propellant has not been damaged, and the stress changes linearly with the strain. With the continuous loading of the load, the dewetting phenomenon begins to appear, and the initial dewetting point is different under different stress states, but generally occurs at the interface between the large-sized AP particles and the matrix; In the stage of damage evolution, the larger stress point of the matrix is also torn immediately, and together with the pores generated by dewetting, it continuously converges into cracks. The appearance of cracks greatly changes the stress distribution of the microstructure of the propellant, and the tip of the crack becomes the main area of stress concentration, which accelerates the propagation of the crack. With the continuous expansion of cracks, the stress is gradually differentiated, and the load on the particles is gradually transferred to the matrix. With the continuous application of the load, The crack begins to expand along a certain direction. Under 1:1 biaxial tensile loading, the crack expands along the diagonal direction, while under non-equi-proportional biaxial tensile loading, the crack expands along the direction perpendicular to the resultant force; finally, in the fracture stage, the propellant loses its bearing capacity due to the formation of penetrating cracks, resulting in complete failure. Compared with uniaxial stretching, the elongation under biaxial tensile loading decreased significantly, and the dewetting point moved forward significantly, among which the maximum elongation and dewetting point elongation under 1:2 biaxial tensile loading decreased most significantly, about 50% and 66% of that in uniaxial tension, respectively, which is similar to the conclusion of the mechanical properties test.

**Figure 19:**
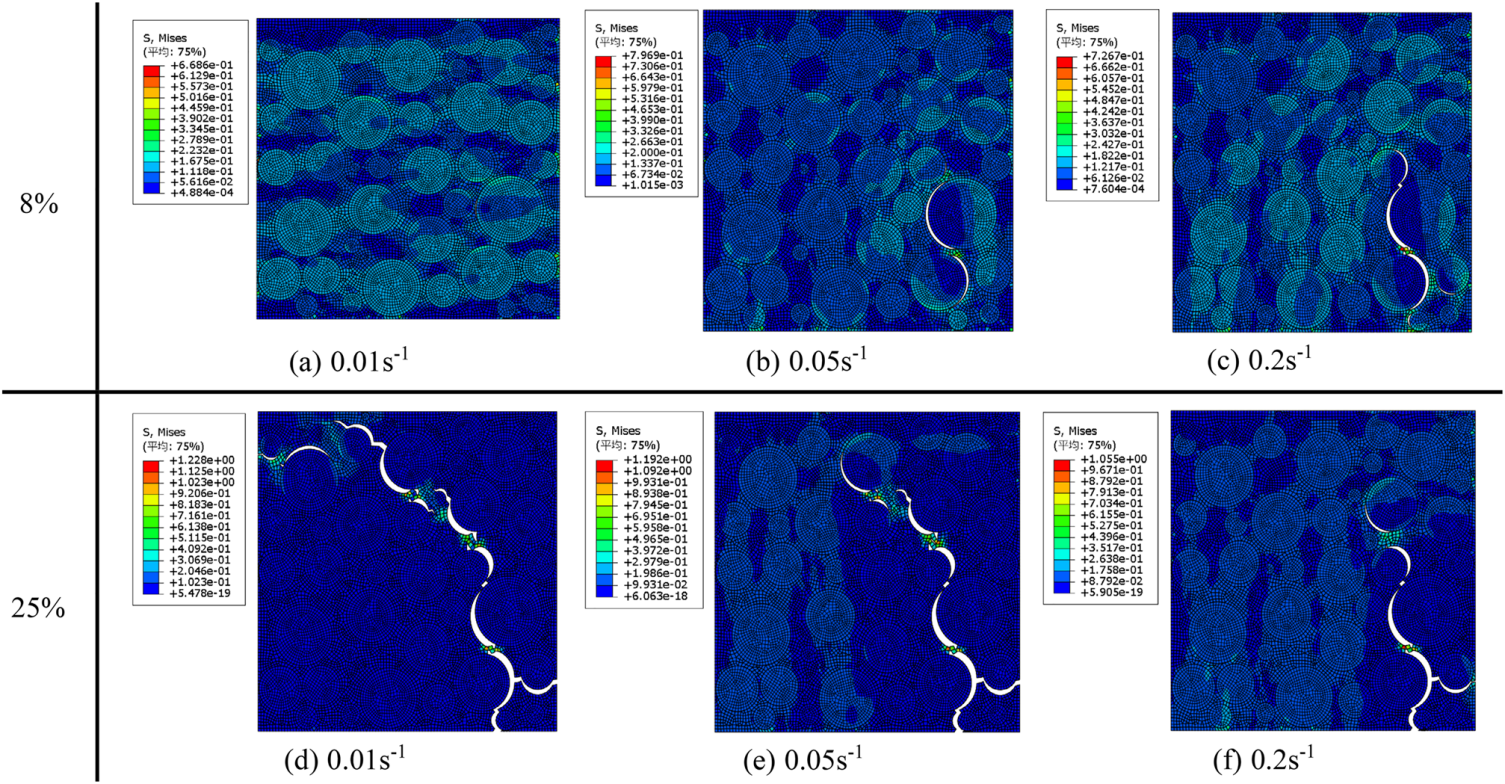
Stress nephograms of different strain rates at 8% and 25% strain under 1:2 biaxial tensile loading.

In order to compare the damage evolution process of the propellant under different strain rates, the stress nephograms under biaxial 1:2 loading and when the strain reaches 8% and 25% are selected for analysis. The results are shown in Fig. 19. It can be clearly seen that the damage degree of the propellant at the same strain varies greatly under different strain rate loadings. When the strain reaches 8%, the propellant is still in the initial linear stage without dewetting under the loading of 0.01 s^−1^ strain rate, while under the loading of 0.05 s^−1^ strain rate, particle dewetting occurs, when the strain rate is further increased to At 0.2 s^−1^, a larger area of dewetting occurred, which indicates that dewetting was more likely to occur under high strain rate loading , it is consistent with the experimental conclusion of macroscopic mechanical properties. However, when the strain reaches 25%, the degree of particle dewetting and matrix fracture of the propellant will be higher at low strain rate. This is because the loading time of the high strain rate is very short, so the interface between the AP particles and the substrate breaks due to the concentrated stress at the crack tip before it can be dewetting, resulting in a small degree of dewetting, which is the main reason for the better mechanical properties of the propellant under high strain rate loading in the macroscopic mechanical properties experiment.

## Conclusion

In this paper, the biaxial stretching experiment of composite solid propellant with variable proportion is realized for the first time through the self-designed biaxial tensile jig and test piece, and the rate dependence and stress state dependence of the mechanical properties of HTPB propellant are analyzed through the experimental results , and then analyzed the damage evolution process of propellant under different strain rates and loading conditions based on ABAQUS simulation software, and compared the results with the traditional uniaxial tensile method. The results show that:

(1) Compared with uniaxial tensile loading, the maximum tensile strength of propellant under biaxial tensile loading will be slightly improved, the maximum strength ratio of the two is 1.0 ~ 1.1, but the maximum elongation will decrease significantly, which is generally 45–85% of uniaxial stretching. Among them, the mechanical properties of the propellant under 1:2 biaxial tensile loading decreased most obviously. With the continuous increase of the loading ratio, the mechanical properties of the propellant under biaxial loading would gradually approach uniaxial stretching.

(2) The effect of strain rate on the mechanical properties of propellant under biaxial tensile loading is similar to that in uniaxial stretching. With the increase of strain rate, the maximum tensile strength and maximum elongation will increase, and.

the dewetting point will move forward. The law of stress–strain curve is also similar to that of uniaxial stretching, which can be divided into initial linear segment, damage evolution stage and fracture stage.

(3) The calculation results show that the propellant has different stress distributions in different stages of the entire failure process. In the initial linear stage, the AP particles carry most of the load, and the matrix is basically unstressed; In the damage evolution stage, due to particle dewetting and matrix fracture, the stress gradually begins to differentiate, the load on AP particles is gradually transferred to the matrix, and the crack tip becomes the stress concentration area and promotes the crack propagation in the direction perpendicular to the resultant force. The propellant dewet more easily under high strain rate loading, but the degree of dewetting is lower when the same strain is reached.

## Data Availability

The data that support the findings of this study are available from the corresponding author upon reasonable request.

## References

[1] Li Z (2005). Investigation of the Work Process of Solid Rocket Motor under Lateral Overload[D].

[2] Gligorijević N, Živković S, Subotić S, Rodić V, Gligorijević I (2015). Effect of cumulative damage on rocket motor service life[J]. J. Energ. Mater..

[3] Chen RX (1991). Design and Research of Solid Rocket Motor[M].

[4] Chyuan S (2002). Nonlinear thermoviscoelastic analysis of Solid propellant grains subjected to temperature loading[J]. Finite Elem. Anal. Des..

[5] Chyuan S (2003). Synamic analysis of solid propellant grains subjected to ignition pressuization loading[J]. J. Sound Vib..

[6] D'Andrea B, Lillo F, MarcellI G (2005). High Speed Mechanical Characterization and Temperature Constraints of Propellants With Energetic Binders[R].

[7] Bills KW, Wiegand JH (1964). Relation of mechanical properties to solid rocket motor failure[J]. Rubber Chem. Technol..

[8] Wang Z, Qiang HF, Wang G (2016). A new test method to obtain biaxial tensile behaviors of solid propellant at high strain rates[J]. Iran. Polym. J..

[9] Liu C, Qiang HF, Wang ZJ (2018). Strength criterion of aged HTPB propellant at low temperature under dynamic loading[J]. J. Propuls. Technol..

[10] Zhao WC, Hang KX, Xu WC (2018). Uniaxial and quasi-biaxial tensile mechanical properties of aged HTPB propellant at low temperatures under dynamic loading[J]. J. Solid Rocket Technol..

[11] Qiang, H. F., Wang, T. J., et al. Failure mechanism, simulation characterization and optimization design of solid rocket motor charge[R]. 973–61338, 2005–2010.

[12] Jia YG, Zhang WH, Zhang W (2011). Optimal design and examination study of biaxial tensile specimens for solid propellant[J]. J. Propuls. Technol..

[13] Jalocha D, Constantinescu A, Nevière R (2015). Prestrained biaxial DMA investigation of viscoelastic nonlinearities in highly filled elastomers[J]. Polym. Testing.

[14] Mcdonald BA, Rice JR, Kirkham MW (2014). Humidity induced burning rate degradation of an iron oxide catalyzed ammonium perchlorate/HTPB composite propellant[J]. Combust. Flame.

[15] Rae PJ, Palmer SJP, Goldrein HT (2002). Quasi-static studies of the deformation and failure of PBX 9501[J]. Proc. R. Soc. A Math. Phys. Eng. Sci..

[16] Rae PJ, Goldrein HT, Palmer SJP (2002). Quasi-static studies of the deformation and failure of β-HMX based polymer bonded explosives[J]. Proc. R. Soc. A.

[17] Collins, B., Maggi, F., Matous, K., et al. Using Tomography to Characterize Heterogeneous Propellants: 46th AIAA Aerospace Sciences Meeting and Exhibit[C], 2008.

[18] Collins BC, Matous K, Rypl D (2010). Three-dimensional reconstruction of statistically optimal unit cells of multimodal particulate composites[J]. Int. J. Multiscale Comput. Eng..

[19] Barenblatt GI (1959). Equilibrium cracks formed during brittle fracture rectilinear cracks in plane plates[J]. J. Appl. Math. Mech..

[20] Dugdale DS (1960). Yielding of steel sheets containing slits[J]. J. Mech. Phys. Solids.

[21] Zhi SJ, Sun B, Zhang JW (2012). Multiscale modeling of heterogeneous propellants from particle packing to grain failure using a surface-based cohesive approach[J]. Acta. Mech. Sin..

[22] Han B, Ju Y (2012). Simulation of crack propagation in HTPB propellant using cohesive zone model[J]. Eng. Fail. Anal..

[23] Tan H, Huang Y, Liu C (2007). The uniaxial tension of particulate composite materials with nonlinear interface debonding[J]. Int. J. Solids Struct..

[24] Francqueville FD, Diani J, Gilormini P (2021). Use of a micromechanical approach to understand the mechanical behavior of solid propellants[J]. Mech. Mater..

[25] Geng TJ, Qiang HF, Wang ZJ (2021). Design of biaxial compression specimen for HTPB composite solid propellant under dynamic loading[J]. Chin. J. Energ. Mater..

[26] Ferron G, Kinde M (1988). JTEV A..

[27] Bhatnagar N, Bhardwaj R, Selvakumar P, Brieu M (2006). Development of a biaxial tensile test fixture for reinforced thermoplastic composites[J]. Polym. Test..

[28] Lu XH (2007). Strength Criteria Investigation and Biaxial Tensile Experiment of 2-axial Fiber-reinforced Composites[D].

[29] Yi ZX (2012). Experimental Study Based on Rubber Stretch[D].

[30] Azoug A, Thorin A (2013). Influence of orthogonal prestrain on the viscoelastic behaviour of highly-filled elastomers[J]. Polym. Test. Lond..

[31] Pan B, Qian K, Xie H (2009). Two-dimensional digital image correlation for in-plane displacement and strain measurement: A review. Meas. Sci. Technol..

[32] Blaber J, Adair B, Antoniou A (2015). Ncorr: Open-source 2D digital image correlation matlab software[J]. Exp. Mech..

[33] Wang Z, Qiang H, Wang G (2015). Tensile mechanical properties and constitutive model for HTPB propellant at low temperature and high strain rate[J]. J. Appl. Polym. Sci..

[34] Wang ZJ, Qiang HF, Wang G (2015). Experimental investigation on high strain rate tensile behaviors of HTPB propellant at low temperatures. Propellants Explos. Pyrotech..

[35] Zhang LH, Cai J (1995). Preliminary study on biaxial tensile test of solid propellant[J]. Chin. J. Explos. Propellants.

[36] Miller TC (2019). Damage and dilatometry for solid propellants with digital image correlation[J]. Propellants Explos. Pyrotech..

[37] Francis EC, Carlton CH (1969). Some aspects of nonlinear mechanical behavior of a composite propellant[J]. J. Spacecr. Rockets.

[38] Zhang, L., Zhi, S. J., Shen, Z. B., et al. (2022) Analysis and verification of rate-dependent damage mechanism of HTPB composite solid propellant[J/OL]. J. Propuls. Technol. 1–12.

[39] Palmer SJP, Field JE, Huntley JM (1993). Deformation, strengths and strains to failure of polymer bonded explosives. Proc R Soc Lond. Math. Phys. Eng. Sci..

[40] Barenblatt GI (1962). The mathematical theory of equi-librium cracks in brittle fracture[J]. Adv. Appl. Mech..

[41] Shiqi Li (2021). Research on the Meso-damage Evolution Law of Solid Motor Grain[D].

[42] Zhou Q-C, Ju Y-T, Wei Z, Han B, Zhou C-S (2014). Cohesive zone modeling of propellant and insulation interface debonding[J]. J. Adhesi..

[43] Han B, Ju YT, Zhou CS (2012). Cohesive zone modeling of propellant and insulation interface debonding. Eng. Fail. Anal..

